# A case–control analysis of oral contraceptive use and breast cancer subtypes in the African American Breast Cancer Epidemiology and Risk Consortium

**DOI:** 10.1186/s13058-015-0535-x

**Published:** 2015-02-21

**Authors:** Traci N Bethea, Lynn Rosenberg, Chi-Chen Hong, Melissa A Troester, Kathryn L Lunetta, Elisa V Bandera, Pepper Schedin, Laurence N Kolonel, Andrew F Olshan, Christine B Ambrosone, Julie R Palmer

**Affiliations:** Slone Epidemiology Center at Boston University, 1010 Commonwealth Avenue, Boston, MA 02215-1201 USA; Department of Cancer Prevention and Control, Roswell Park Cancer Institute, Elm & Carlton Streets, Buffalo, NY 14263 USA; Department of Epidemiology, University of North Carolina at Chapel Hill, 135 Dauer Drive, McGavran-Greenberg Hall, Campus Box #7435, Chapel Hill, NC 27599 USA; Department of Biostatistics, Boston University School of Public Health, Crosstown Center, 801 Massachusetts Avenue, 3rd Floor, Boston, MA 02118 USA; Cancer Prevention and Control Program, Rutgers Cancer Institute of New Jersey, Rutgers, The State University of New Jersey, 195 Little Albany Street, New Brunswick, NJ 08903 USA; Department of Cell, Developmental & Cancer Biology, Oregon Health & Science University, Rm RJH 5518 Richard Jones Hall/Mail Code L215, 3181 SW Sam Jackson Park Road, Portland, OR 97239 USA; Cancer Epidemiology Program, University of Hawai’i Cancer Center, 701 Ilalo Street, Honolulu, HI 96813 USA

## Abstract

**Introduction:**

Recent oral contraceptive (OC) use has been consistently associated with increased risk of breast cancer, but evidence on specific breast cancer subtypes is sparse.

**Methods:**

We investigated recency and duration of OC use in relation to molecular subtypes of breast cancer in a pooled analysis of data from the African American Breast Cancer Epidemiology and Risk Consortium. The study included 1,848 women with estrogen receptor-positive (ER+) breast cancer, 1,043 with ER-negative (ER-) breast cancer (including 494 triple negative (TN) tumors, which do not have receptors for estrogen, progesterone, and human epidermal growth factor 2), and 10,044 controls. Multivariable polytomous logistic regression models were used to estimate odds ratios (ORs) and 95% confidence intervals (CIs) for exposure categories relative to never use, controlling for potential confounding variables.

**Results:**

OC use within the previous 5 years was associated with increased risk of ER+ (OR 1.46, 95% CI 1.18 to 1.81), ER- (OR 1.57, 95% CI 1.22 to 1.43), and TN (OR 1.78, 95% CI 1.25 to 2.53) breast cancer. The risk declined after cessation of use but was apparent for ER+ cancer for 15 to 19 years after cessation and for ER- breast cancer for an even longer interval after cessation. Long duration of use was also associated with increased risk of each subtype, particularly ER-.

**Conclusions:**

Our results suggest that OC use, particularly recent use of long duration, is associated with an increased risk of ER+, ER-, and TN breast cancer in African American women. Research into mechanisms that explain these findings, especially the association with ER- breast cancer, is needed.

## Introduction

A combined analysis of 54 studies observed a 24% increase in breast cancer risk for current use of oral contraceptives (OCs) and a 16% increase in risk for <5 years since stopping OC use [1]; the increase largely disappeared within 10 years after cessation. However, the length of time during which risk remains elevated is uncertain because few studies have assessed long intervals since stopping [2,3]. Subsequent studies suggest that long duration of OC use may also increase incident breast cancer risk [4-13]. These studies were based primarily on data from White women.

Gene expression studies have identified at least five intrinsic subtypes of breast cancer, and the most common clinical markers of breast cancer heterogeneity to date have been presence or absence of estrogen receptors (ERs) or progesterone receptors (PRs) and overexpression of human epidermal growth factor receptor 2 (HER2) [14]. Tumors that are ER– or triple negative (TN, defined as ER–/PR–/HER2–) have a lower 5-year survival than ER+ tumors [15,16], and are more common in African American women than in women of European ancestry [17-19].

Some risk factors, particularly hormone-related factors, appear to differentially associate with specific breast cancer subtype [20,21], but evidence on OC use in relation to specific breast cancer subtypes is sparse and the findings are inconsistent [22]. Some studies find OC use to be associated with increased risk of ER+ and ER– breast cancer [4,9], others with increased risk of ER– breast cancer only [23], and yet others observe an association solely for ER+ breast cancer [24]. With respect to TN breast cancer, null results [2,25] and an increased risk [23,26] have been observed for OC use.

The objective of the present study was to investigate OC use in relation to molecular subtypes of breast cancer in African American women using data from a large consortium, the African American Breast Cancer Epidemiology and Risk (AMBER) project. In particular, we were able to assess long duration of OC use and recency of use, including intervals as long as 30 years or more since last use.

## Methods

### Participating studies and data collection

The AMBER Consortium has been described in detail elsewhere [27]. Its purpose is to provide enough data and samples for informative assessment of genetic and nongenetic factors in relation to breast cancer subtypes in African American women. The AMBER Consortium pools data from two large case–control studies, the Carolina Breast Cancer Study (CBCS) and the Women’s Circle of Health Study (WCHS), and two large cohort studies, the Black Women’s Health Study (BWHS) and the Multiethnic Cohort Study. Owing to missing data on recency of OC use, the Multiethnic Cohort Study was not included in this analysis, whereas data from the BWHS, the CBCS, and the WCHS were included.

The BWHS is a prospective cohort study of 59,000 African American women aged 21 to 69 years at baseline in 1995 [28]. Participants report data on OC use, other risk factors, and incident disease on biennial questionnaires. Questionnaire follow-up is complete for 80% of the baseline cohort through 2011. New diagnoses of cancer are self-reported on the follow-up questionnaires or identified through linkage with state cancer registries [9,27]. For each case, controls were selected from the pool of BWHS participants who had not developed breast cancer at the date of the case’s breast cancer diagnosis (index date). Controls were frequency-matched to cases on 5-year age group, geographic region, and most recent questionnaire completed before the matched case diagnosis. Exposure and covariate data from that questionnaire and preceding questionnaires were used for the analysis.

The CBCS is a population-based case–control study of women aged 20 to 74 years in North Carolina conducted from 1993 through 2001 [29]. Breast cancer cases are identified through the North Carolina Central Cancer Registry. We used data from the first two phases, in which younger and African American cases were oversampled. Cases and controls were sampled using a modification of randomized recruitment. Controls younger than age 65 years were identified from Division of Motor Vehicle lists and controls age 65 years or older were identified from Health Care Financing Administration lists. Controls were frequency-matched to cases on age in 5-year age groups. Exposure and covariate data were collected through in-person interviews with reference to the year before diagnosis (cases) or interview date (controls) [29]. Response rates were 76% for cases and 55% controls for recruitment of invasive breast cancer cases and matched controls, while response rates were 83% for cases and 65% controls for recruitment of *in situ* breast cancer cases and matched controls [29].

The WCHS is a population-based case–control study of women aged 20 to 75 years that began in New York in 2003 and currently enrolls participants in New Jersey only [30,31]. Breast cancer cases were identified through major hospitals in New York City and through the New Jersey Cancer Registry. Controls were identified through random digit dialing and through community-based recruitment and are frequency matched to cases on age in 5-year age groups [31]. Exposure and covariate data were collected through in-person interviews with reference to the year before diagnosis (cases) or interview date (controls) [30]. Response rates were 78.7% in cases and 48.2% in controls [32].

Each study obtained informed consent from all participants and was approved by the relevant Institutional Review Boards, which are listed in Acknowledgements. Each study prepared a data file that included questionnaire data for a specified set of variables and pathology data from medical records or state cancer registry records. Data were harmonized at the data coordinating center with regular input from each study’s principal investigator and data collection staff.

### Cases

Incident cases of breast cancer (primary site codes C500 to C506, C508, and C509 in the *International Classification of Diseases for Oncology*, Third Edition), not including lobular carcinoma *in situ* (8520/2), phyllodes tumors (9020), or Paget’s disease (8540/3), were confirmed through medical records and state cancer registries [33]. Immunohistochemistry results were obtained from hospital pathology records and cancer registry data, and were used to classify cases as ER+, ER–, and TN (ER*–/*PR*–*/HER2–). ER data were available for 72% of cases and PR data were available for 69% of cases. Testing for HER2 has become routine more recently; therefore, HER2 data were available for only 49% of cases. There were no statistically significant differences between women with and without known receptor status by age or OC use. The proportion of ER– cases (Table 1) are similar to those observed for African American women in other data sources [34-36].

**Table 1:** **Characteristics of cases and controls in the AMBER Consortium, by included study (BWHS, CBCS, WCHS)**

	**BWHS**		**CBCS**		**WCHS**		**Total**	
	**Cases**	**Controls**	**Cases**	**Controls**	**Cases**	**Controls**	**Cases**	**Controls**
**Characteristics of breast cancer cases**								
Total	1,313		804		774		2,891	
ER+	862 (66)		436 (54)		550 (71)		1,848 (64)	
ER–	451 (34)		368 (46)		224 (29)		1,043 (36)	
Triple negative	155 (12)		210 (26)		129 (17)		494 (17)	
Age at diagnosis								
< 40 years	98 (8)		129 (16)		87 (11)		324 (11)	
40 to 49 years	385 (29)		262 (33)		209 (27)		856 (30)	
50 to 59 years	456 (35)		190 (24)		277 (36)		923 (32)	
≥ 60 years	374 (29)		223 (28)		191 (25)		788 (27)	
**Oral contraceptive use among cases and controls**								
Ever use								
No	525 (40)	3,768 (45)	392 (49)	387 (49)	374 (48)	477 (49)	1,291 (45)	4,632 (46)
Yes	788 (60)	4,522 (55)	412 (51)	398 (51)	400 (52)	492 (51)	1,600 (55)	5,412 (54)
Recency of OC use								
< 5 years ago	182 (14)	950 (11)	68 (8)	52 (7)	64 (8)	73 (8)	314 (11)	1,075 (11)
5 to 9 years ago	68 (5)	464 (6)	44 (5)	32 (4)	36 (5)	48 (5)	148 (5)	544 (5)
10 to 14 years ago	77 (6)	499 (6)	53 (7)	59 (8)	39 (5)	37 (4)	169 (6)	595 (6)
15 to 19 years ago	105 (8)	620 (7)	95 (12)	85 (11)	45 (6)	52 (5)	245 (8)	757 (8)
20 to 24 years ago	108 (8)	692 (8)	90 (11)	102 (13)	47 (6)	65 (7)	245 (8)	859 (9)
25 to 29 years ago	124 (9)	653 (8)	35 (4)	46 (6)	55 (7)	79 (8)	214 (7)	778 (8)
≥ 30 years ago	124 (9)	644 (8)	24 (3)	21 (3)	108 (14)	130 (13)	256 (9)	795 (8)
Duration of OC use								
< 5 years	316 (24)	1,934 (23)	159 (20)	174 (22)	152 (20)	207 (21)	627 (22)	2,315 (23)
5 to 9 years	235 (18)	1,364 (17)	125 (16)	123 (16)	121 (16)	142 (15)	481 (17)	1,629 (16)
10 to 14 years	178 (14)	874 (11)	81 (11)	61 (8)	71 (9)	75 (8)	330 (11)	1,010 (10)
≥ 15 years	59 (4)	350 (4)	47 (6)	40 (5)	56 (7)	68 (7)	162 (6)	458 (5)

Data presented as *n* (%). Total percentages may not add up to 100% due to rounding. Excluding women with unknown duration of OC use and unknown ER status. ER status was unknown for 1,151 breast cancer cases: 90 in CBCS, 308 in WCHS, and 753 in BWHS. AMBER, African American Breast Cancer Epidemiology and Risk; BWHS, Black Women’s Health Study; CBCS, Carolina Breast Cancer Study; ER, estrogen receptor; OC, oral contraceptive; WCHS, Women’s Circle of Health Study.

### Exposure variables

#### Oral contraceptive use

The main exposures of interest for the present analyses were self-reported recency of OC use (years since last use) and duration of OC use. Women with a total duration of OC use of less than 1 year were considered to be never users. Recency of OC use was categorized as within the previous 5 years, 5 to 9 years ago, 10 to 14 years ago, 15 to 19 years ago, 20 to 24 years ago, 25 to 29 years ago, or ≥30 years ago. Duration of OC use was categorized as 1 to 4 years, 5 to 9 years, 10 to 14 years, or ≥15 years.

#### Covariates

Self-reported data were available on the following covariates: history of breast cancer in a first-degree female relative (mother, sister, or daughter), age at menarche, parity, age at first birth, lifetime duration of breastfeeding, menopausal status, age at menopause, menopausal female hormone use, height, weight, educational attainment, alcohol consumption, and cigarette smoking. For menopausal status, women who were still menstruating were classified as premenopausal. Women who had experienced a natural menopause or a hysterectomy with bilateral oophorectomy were classified as postmenopausal. Also classified as postmenopausal were women (*n* = 60) whose periods had stopped due to use of mediation, radiation, or chemotherapy for various health conditions other than breast cancer. Women who had a hysterectomy with retention of one or both ovaries were classified as premenopausal if their index age was less than or equal to the age at which 10% of women in their study had reached natural menopause, and as postmenopausal if their index age was greater than or equal to the age at which 90% of women in their study had reached natural menopause; otherwise, these participants were classified as having unknown menopausal status.

### Data analysis

Polytomous logistic regression models were used to calculate odds ratios (ORs) and 95% confidence intervals (CIs) for the relation of measures of OC use to ER+ and ER– breast cancer risk. For TN breast cancer risk, polytomous models had three outcomes: TN, ER+, and ER–/not TN. We controlled for age (continuous), study (CBCS, WCHS, BWHS), time period of case diagnosis/control index date (1993 to 1998, 1999 to 2005, 2006 to 2013), family history of breast cancer (yes, no), age at menarche (<12, 12 to 13, ≥14 years), parity (0, 1, 2, ≥3 births), age at first birth (<20, 20 to 24, ≥25 years), lactation (never, ever), menopausal status and age at menopause (premenopausal, <45, 45 to 49, 50 to 54, ≥55 years), female hormone use (0, <5, ≥5 years of use), body mass index (weight in kilograms divided by height in meters squared; <18.5, 18.5 to 24.9, 25.0 to 29.9, 30.0 to 34.9, ≥35.0 kg/m^2^), educational attainment (<12, 12, 13 to 15, 16, >16 years), alcohol consumption (<1, 1 to 6, ≥7 drinks per week), smoking status (0, <10, 10 to 19, ≥20 pack-years), and geographic region (New Jersey, other Northeast, South, Midwest, West). We used the missing indicator method to handle missing data. For menopausal status, 8.8% of women had missing data; for each of the other covariates, fewer than 2% had missing data. To test for trend across categories of OC duration or OC recency, the ordinal variable was treated as a continuous variable in the regression model. Trend tests were carried out among OC users only. To examine potential differences by subtype, we carried out case–case analyses, in which ORs were computed for the relation of OC use to ER– relative to ER+ breast cancer. Effect modification was explored through stratification by age, menopausal status, body mass index, and parity. Interaction on the multiplicative scale was tested by the likelihood ratio test, comparing models with and without multiplicative interaction terms. Analyses were performed using SAS 9.2 statistical software (SAS Institute Inc., Cary, NC, USA).

In addition to the main analyses, which were carried out in pooled data, study-specific ORs were calculated and combined in a random-effects meta-analysis. The Cochran’s Q statistic was used to test for heterogeneity [37]. Meta-analyses were performed using Stata/SE 11.2 statistical software (StataCorp LP, College Station, TX, USA).

## Results

Characteristics of cases and controls are presented in Table 1. Among the 2,891 cases, 1,043 (36%) had tumors that were classified as ER– and 1,848 (64%) were classified as ER+ (Table 1). In addition, 494 of the ER– cases were TN breast cancer. The mean age at diagnosis was 52.7, with 11.2% of cases diagnosed before age 40 and 27.3% diagnosed at age 60 or older. OC use was similar across the three participating studies.

Table 2 presents analyses of OC use in relation to ER+ and ER– breast cancer. Ever OC use was positively associated with both ER+ (OR = 1.15, 95% CI = 1.02 to 1.28) and ER– (OR = 1.24, 95% CI = 1.07 to 1.43) breast cancer. There was a significant dose–response association with recency of OC use for both ER+ and ER– breast cancer (*P* trend <0.01 and *P* trend = 0.05, respectively). The strongest associations were for OC use within the previous 5 years (OR = 1.46, 95% CI = 1.18 to 1.81 for ER+ breast cancer and OR = 1.57, 95% CI = 1.22 to 2.03 for ER– breast cancer). For ER+ breast cancer, the ORs were elevated through 15 to 19 years since last use and the estimate for 15 to 19 years was statistically significant (OR = 1.29, 95% CI = 1.04 to 1.58). For ER– breast cancer, the pattern was less consistent; the OR declined to 1.07 (95% CI =, 0.83 to 1.37) at 20 to 24 years since last use, but there was a statistically significant elevation at 25 to 29 years since last use (OR = 1.41, 95% CI = 1.09 to 1.81). Risk of ER– cancer increased with increasing duration of OC use, with ORs of 1.43 (95% CI = 1.13 to 1.80) and 1.54 (95% CI = 1.15 to 2.06) for 10 to 14 and ≥15 years duration, respectively. For ER+ breast cancer, the OR for 10 to 14 years duration was elevated (1.35, 95% CI = 1.12 to 1.61) but the estimate for ≥15 years was approximately 1.00. Among recent users, risk increased with increasing duration of OC use for both ER+ and ER– cancer. The OR for ≥10 years of use that continued into the 5 years before diagnosis or index date was 1.65 (95% CI = 1.27 to 2.15) for ER+ cancer and 1.73 (95% CI = 1.26 to 2.37) for ER– cancer. The corresponding estimates for ≥10 years of use that ended at least 20 years previously was 0.88 (95% CI = 0.65 to 1.19) for ER+ cancer and 1.50 (95% CI = 1.03 to 2.17) for ER– cancer.

**Table 2:** **Oral contraceptive use in relation to ER+ and ER– breast cancer** ^**a**^

			**ER+**			**ER–**		
		**Controls (** ***n*** **)**	**Cases (** ***n*** **)**	**OR**	**(95% CI)**	**Cases (** ***n*** **)**	**OR**	**(95% CI)**
Never users		4,632	862	1.00	Reference	429	1.00	Reference
Ever users		5,415	990	1.15	(1.02 to 1.28)	615	1.24	(1.07 to 1.43)
OC recency								
< 5 years ago		1,075	183	1.46	(1.18 to 1.81)	131	1.57	(1.22 to 2.03)
5 to 9 years ago		544	84	1.16	(0.89 to 1.52)	64	1.33	(0.97 to 1.82)
10 to 14 years ago		595	105	1.25	(0.98 to 1.60)	64	1.12	(0.82 to 1.52)
15 to 19 years ago		757	153	1.28	(1.04 to 1.58)	92	1.14	(0.88 to 1.48)
20 to 24 years ago		859	148	1.01	(0.82 to 1.24)	97	1.07	(0.83 to 1.37)
25 to 29 years ago		781	123	0.93	(0.75 to 1.15)	91	1.41	(1.09 to 1.81)
≥ 30 years ago		795	185	1.14	(0.94 to 1.38)	71	1.14	(0.85 to 1.51)
*P* trend				<0.01			0.05	
OC duration								
< 5 years		2,315	367	1.03	(0.89 to 1.19)	260	1.21	(1.01 to 1.44)
5 to 9 years		1,629	321	1.25	(1.07 to 1.46)	160	1.07	(0.87 to 1.32)
10 to 14 years		1,010	209	1.35	(1.12 to 1.61)	121	1.43	(1.13 to 1.80)
≥ 15 years		458	89	0.98	(0.76 to 1.27)	73	1.54	(1.15 to 2.06)
*P* trend				0.13			0.05	
Joint OC exposure (recency and duration)								
Since last use	Duration							
< 5 years	< 5 years	329	39	1.13	(0.78 to 1.62)	30	1.20	(0.78 to 1.83)
	5 to 9 years	289	45	1.60	(1.12 to 2.28)	33	1.67	(1.10 to 2.53)
	≥ 10 years	457	99	1.65	(1.27 to 2.15)	68	1.73	(1.26 to 2.37)
5 to 9 years	< 5 years	172	22	1.21	(0.75 to 1.94)	16	1.26	(0.72 to 2.21)
	5 to 9 years	140	30	1.62	(1.05 to 2.52)	13	0.94	(0.51 to 1.74)
	≥ 10 years	232	32	0.92	(0.62 to 1.38)	35	1.59	(1.06 to 2.38)
10 to 19 years	< 5 years	466	66	1.09	(0.82 to 1.47)	58	1.21	(0.87 to 1.67)
	5 to 9 years	451	87	1.33	(1.02 to 1.73)	46	0.94	(0.66 to 1.33)
	≥ 10 years	435	105	1.41	(1.11 to 1.81)	52	1.24	(0.90 to 1.72)
≥ 20 years	< 5 years	1,344	238	1.02	(0.86 to 1.20)	155	1.23	(1.00 to 1.51)
	5 to 9 years	748	157	1.13	(0.92 to 1.38)	65	1.00	(0.75 to 1.33)
	≥ 10 years	340	61	0.88	(0.65 to 1.19)	39	1.50	(1.03 to 2.17)

CI, confidence interval; ER, estrogen receptor; OC, oral contraceptive; OR, odds ratio. ^a^ORs adjusted for age, study, time period, geographic region, education, age at menarche, parity, age at first birth, lactation, first-degree family history of breast cancer, menopausal status and age at menopause, duration of female hormone use, body mass index, alcohol consumption, and pack-years of cigarette smoking. *P* trend does not include never users.

In a case–case analysis (data not shown), ever use of OCs (OR = 1.09, 95% CI = 0.92 to 1.29) and OC use within the previous 5 years (OR = 1.08, 95% CI = 0.79 to 1.46) were not associated with ER– breast cancer, relative to ER+ breast cancer. Increased risk of ER– breast cancer was associated with long duration (≥15 years) of OC use relative to ER+ cancer: the OR was 1.51 (95% CI = 1.07 to 2.15).

In an analysis stratified by invasive and *in situ* breast cancer (data not shown), results for invasive cancer (1,150 ER+ and 629 ER– cases) were similar to the overall findings: the ORs for invasive ER+ cancer were 1.14 (95% CI = 1.00 to 1.31) for ever use, 1.41 (95% CI = 1.10 to 1.81) for use within the previous 5 years, and 0.95 (0.69 to 1.30) for duration of 15 years or more; the corresponding estimates for invasive ER– cancer were 1.36 (95% CI = 1.14 to 1.62), 1.62 (95% CI = 1.20 to 2.20) and 1.59 (1.11 to 2.29), respectively. For *in situ* cancer, based on 255 ER+ cases, risk of ER+ breast cancer was significantly elevated for recent OC use (OR = 1.66, 95% CI = 1.02 to 2.68), but was not associated with long duration of OC use (OR = 0.95, 95% CI = 0.51 to 1.75). There were too few ER– *in situ* cases (*n* = 43) for an informative analysis.

The relation of OC use to risk of TN breast cancer is presented in Table 3. The OR for recent use of OCs relative to never use was 1.78 (95% CI = 1.25 to 2.53) and risk declined as the interval since last use increased (*P* trend =0.05). Risk increased with increasing duration of OC use, with an OR of 1.62 (95% CI = 1.11 to 2.38) for ≥15 years of use (*P* trend = 0.05). Analyses of a joint variable for duration and recency of use indicated that the increased risk associated with at least 10 years of OC use was statistically significant both for use less than 5 years ago (OR = 1.66, 95% CI = 1.06 to 2.00) and use 5 to 9 years ago (OR = 1.79, 95% CI = 1.04 to 3.07).

**Table 3:** **Oral contraceptive use in relation to triple-negative breast cancer** ^**a**^

		**Controls (** ***n*** **)**	**Cases (** ***n*** **)**	**OR**	**(95% CI)**
Never users		4,632	213	1.00	Reference
Ever users		5,415	282	1.14	(0.93 to 1.40)
OC recency					
< 5 years ago		1,075	66	1.78	(1.25 to 2.53)
5 to 9 years ago		544	33	1.37	(0.88 to 2.12)
10 to 14 years ago		595	37	0.99	(0.63 to 1.56)
15 to 19 years ago		757	47	1.19	(0.83 to 1.72)
20 to 24 years ago		859	40	0.85	(0.89 to 1.24)
25 to 29 years ago		781	40	1.26	(0.87 to 1.81)
≥ 30 years ago		795	30	0.91	(0.59 to 1.38)
*P* trend				0.05	
OC duration					
< 5 years		2,315	117	1.08	(0.84 to 1.39)
5 to 9 years		1,629	73	0.99	(0.74 to 1.34)
10 to 14 years		1,010	51	1.26	(0.90 to 1.77)
≥ 15 years		458	40	1.62	(1.11 to 2.38)
*P* trend				0.05	
**Joint OC exposure**					
Since last use	Duration				
< 5 years	< 5 years	329	14	1.31	(0.72 to 2.40)
	5 to 9 years	289	21	2.70	(1.58 to 4.61)
	≥ 10 years	457	30	1.66	(1.06 to 2.60)
5 to 9 years	< 5 years	172	5	0.89	(0.35 to 2.30)
	5 to 9 years	140	7	1.08	(0.47 to 2.48)
	≥ 10 years	232	19	1.79	(1.04 to 3.07)
10 to 19 years	< 5 years	466	26	1.12	(0.70 to 1.79)
	5 to 9 years	451	21	0.87	(0.53 to 1.45)
	≥ 10 years	435	27	1.36	(0.87 to 2.11)
≥ 20 years	< 5 years	1,344	72	1.11	(0.83 to 1.49)
	5 to 9 years	748	22	0.67	(0.42 to 1.06)
	≥ 10 years	340	15	1.14	(0.65 to 2.00)

CI, confidence interval; OC, oral contraceptive; OR, odds ratio. ^a^ORs adjusted for age, study, time period, geographic region, education, age at menarche, parity, age at first birth, lactation, first-degree family history of breast cancer, menopausal status and age at menopause, duration of female hormone use, body mass index, alcohol consumption, and pack-years of cigarette smoking. *P* trend does not include never users.

In analyses stratified by age (<40 years, 40 to 49 years, ≥50 years), recent and long-duration OC use were associated with increased risk of breast cancer in every age group (Table 4). The interaction was not statistically significant for OC recency or duration (*P* interaction = 0.75 and *P* interaction = 0.77, respectively).

**Table 4:** **Oral contraceptive use in relation to breast cancer risk, stratified by age** ^**a**^

	**Age <40**				**Age 40 to 49**				**Age ≥50**			
	**Controls (** ***n*** **)**	**Cases (** ***n*** **)**	**OR**	**(95% CI)**	**Controls (** ***n*** **)**	**Cases (** ***n*** **)**	**OR**	**(95% CI)**	**Controls (** ***n*** **)**	**Cases (** ***n*** **)**	**OR**	**(95% CI)**
Never users	351	85	1.00	Reference	1,022	272	1.00	Reference	3,259	934	1.00	Reference
Ever users	796	239	1.33	(0.97 to 1.81)	2,057	584	1.18	(0.98 to 1.42)	2,562	782	1.15	(1.01 to 1.30)
OC recency												
< 5 years ago	482	130	1.55	(1.09 to 2.19)	482	149	1.60	(1.23 to 2.07)	111	35	1.50	(0.99 to 2.28)
5 to 9 years ago	185	53	1.20	(0.77 to 1.86)	292	74	1.18	(0.86 to 1.63)	94	21	0.95	(0.57 to 1.58)
≥ 10 years ago	154	56	1.04	(0.67 to 1.61)	1,282	361	1.07	(0.88 to 1.31)	2,351	716	1.14	(1.00 to 1.29)
*P* trend			0.02				<0.01				0.45	
OC duration												
< 5 years	342	84	1.18	(0.81 to 1.70)	835	225	1.12	(0.90 to 1.40)	1,138	318	1.08	(0.92 to 1.27)
5 to 9 years	239	79	1.40	(0.95 to 2.06)	629	160	1.06	(0.83 to 1.35)	761	242	1.20	(1.00 to 1.44)
≥ 10 years	215	76	1.48	(0.99 to 2.20)	593	199	1.39	(1.10 to 1.76)	660	217	1.20	(0.99 to 1.45)
*P* trend			0.17				0.11				0.26	

CI, confidence interval; OC, oral contraceptive; OR, odds ratio. ^a^ORs adjusted for age, study, time period, geographic region, education, age at menarche, parity, age at first birth, lactation, first-degree family history of breast cancer, menopausal status and age at menopause, duration of female hormone use, body mass index, alcohol consumption, and pack-years of cigarette smoking. *P* trend does not include never users.

In analyses stratified by menopausal status (Table 5), ORs were higher in premenopausal women, but there was not a statistically significant interaction (*P* interaction = 0.16 for recency, *P* interaction = 0.06 for duration). Breast cancer risk was elevated for recent OC use in both nulliparous and parous women (Table 5): among nulliparous women the OR for OC use within the previous 5 years was 1.37 (95% CI = 0.98 to 1.92), and the corresponding OR for parous women was 1.55 (95% CI = 1.26 to 1.90) (*P* interaction = 0.08). Long duration of OC use was associated with breast cancer among parous women, but not among nulliparous women (Table 5). The interaction was not significant (*P* = 0.42).

**Table 5:** **Oral contraceptive use in relation to breast cancer risk, stratified by menopausal status and parity** ^**a**^

	**Controls (** ***n*** **)**	**Cases (** ***n*** **)**	**OR**	**(95% CI)**	**Controls (** ***n*** **)**	**Cases (** ***n*** **)**	**OR**	**(95% CI)**
	**Premenopausal women**				**Postmenopausal women**			
Never users	1,371	363	1.00	Reference	2,903	854	1.00	Reference
Ever users	2,667	767	1.24	(1.05 to 1.45)	2,184	703	1.16	(1.01 to 1.33)
OC recency								
< 5 years ago	937	273	1.54	(1.25 to 1.90)	90	19	1.02	(0.59 to 1.76)
5 to 9 years ago	404	113	1.31	(1.01 to 1.72)	99	21	0.85	(0.50 to 1.44)
≥ 10 years ago	1,324	380	1.08	(0.90 to 1.30)	1,989	654	1.17	(1.02 to 1.34)
*P* trend			<0.01				0.39	
OC duration								
< 5 years	1,066	276	1.11	(0.91 to 1.35)	992	301	1.14	(0.96 to 1.36)
5 to 9 years	818	217	1.14	(0.92 to 1.41)	648	222	1.24	(1.02 to 1.50)
≥ 10 years	782	274	1.53	(1.25 to 1.88)	542	175	1.09	(0.88 to 1.34)
*P* trend			<0.01				0.83	
	**Nulliparous women**				**Parous women**			
Never users	917	244	1.00	Reference	3,706	1,046	1.00	Reference
Ever users	1,230	267	1.09	(0.87 to 1.38)	4,183	1,338	1.21	(1.08 to 1.35)
OC recency								
< 5 years ago	406	93	1.37	(0.98 to 1.92)	669	211	1.55	(1.26 to 1.90)
5 to 9 years ago	160	22	0.77	(0.46 to 1.29)	383	126	1.39	(1.08 to 1.77)
≥ 10 years ago	663	151	1.05	(0.81 to 1.36)	3,123	982	1.15	(1.03 to 1.29)
*P* trend			0.16				<0.01	
OC duration								
< 5 years	438	89	1.06	(0.79 to 1.44)	1,875	538	1.11	(0.97 to 1.27)
5 to 9 years	361	76	1.08	(0.78 to 1.50)	1,268	405	1.22	(1.05 to 1.42)
≥ 10 years	431	102	1.13	(0.84 to 1.52)	1,037	390	1.37	(1.17 to 1.60)
*P* trend			0.61				0.01	

CI, confidence interval; OC, oral contraceptive; OR, odds ratio. ^a^ORs adjusted for age, study, time period, geographic region, education, age at menarche, parity, age at first birth, lactation, first-degree family history of breast cancer, menopausal status and age at menopause, duration of female hormone use, body mass index, alcohol consumption, and pack-years of cigarette smoking, where appropriate. *P* trend does not include never users. Women who were missing menopausal status (*n* = 1,218) were excluded from the analysis that stratified on this variable. Women who were missing parity (*n* = 13) were excluded from the analysis that stratified on this variable.

Recent and long-duration OC use were associated with larger increases in breast cancer risk among women who were overweight or obese than among women with body mass index <25 kg/m^2^ (Table 6). The interaction was significant for recent OC use (*P* interaction = 0.04), but not for duration of OC use (*P* interaction = 0.16).

**Table 6:** **Oral contraceptive use in relation to breast cancer risk, stratified by body mass index** ^**a**^

	**BMI <25.0 kg/m** ^**2**^				**BMI 25.0 to 29.9 kg/m** ^**2**^				**BMI ≥30.0 kg/m** ^**2**^			
	**Controls (** ***n*** **)**	**Cases (** ***n*** **)**	**OR**	**(95% CI)**	**Controls (** ***n*** **)**	**Cases (** ***n*** **)**	**OR**	**(95% CI)**	**Controls (** ***n*** **)**	**Cases (** ***n*** **)**	**OR**	**(95% CI)**
Never users	931	231	1.00	Reference	1,503	387	1.00	Reference	2,096	637	1.00	Reference
Ever users	1,371	350	1.09	(0.88 to 1.35)	1,746	521	1.13	(0.95 to 1.35)	2,211	707	1.26	(1.09 to 1.45)
OC recency												
< 5 years ago	361	87	1.22	(0.87 to 1.71)	320	111	1.58	(1.16 to 2.14)	377	112	1.57	(1.19 to 2.08)
5 to 9 years ago	156	32	0.87	(0.55 to 1.38)	175	46	1.06	(0.71 to 1.58)	204	69	1.71	(1.23 to 2.37)
≥ 10 years ago	851	230	1.09	(0.87 to 1.38)	1,248	363	1.09	(0.90 to 1.31)	1,627	518	1.17	(1.01 to 1.36)
*P* trend			0.17				0.05				0.04	
OC duration												
< 5 years	566	135	1.05	(0.80 to 1.37)	726	198	1.12	(0.90 to 1.39)	986	278	1.09	(0.91 to 1.30)
5 to 9 years	408	109	1.11	(0.83 to 1.49)	544	152	1.04	(0.82 to 1.33)	651	213	1.34	(1.09 to 1.64)
≥ 10 years	397	105	1.11	(0.83 to 1.50)	476	171	1.27	(1.00 to 1.61)	571	212	1.48	(1.20 to 1.82)
*P* trend			0.67				0.48				<0.01	

BMI, body mass index; CI, confidence interval; OC, oral contraceptive; OR, odds ratio. ^a^ORs adjusted for age, study, time period, geographic region, education, age at menarche, parity, age at first birth, lactation, first-degree family history of breast cancer, menopausal status and age at menopause, duration of female hormone use, body mass index, alcohol consumption, and pack-years of cigarette smoking. *P* trend does not include never users.

**Figure 1:**
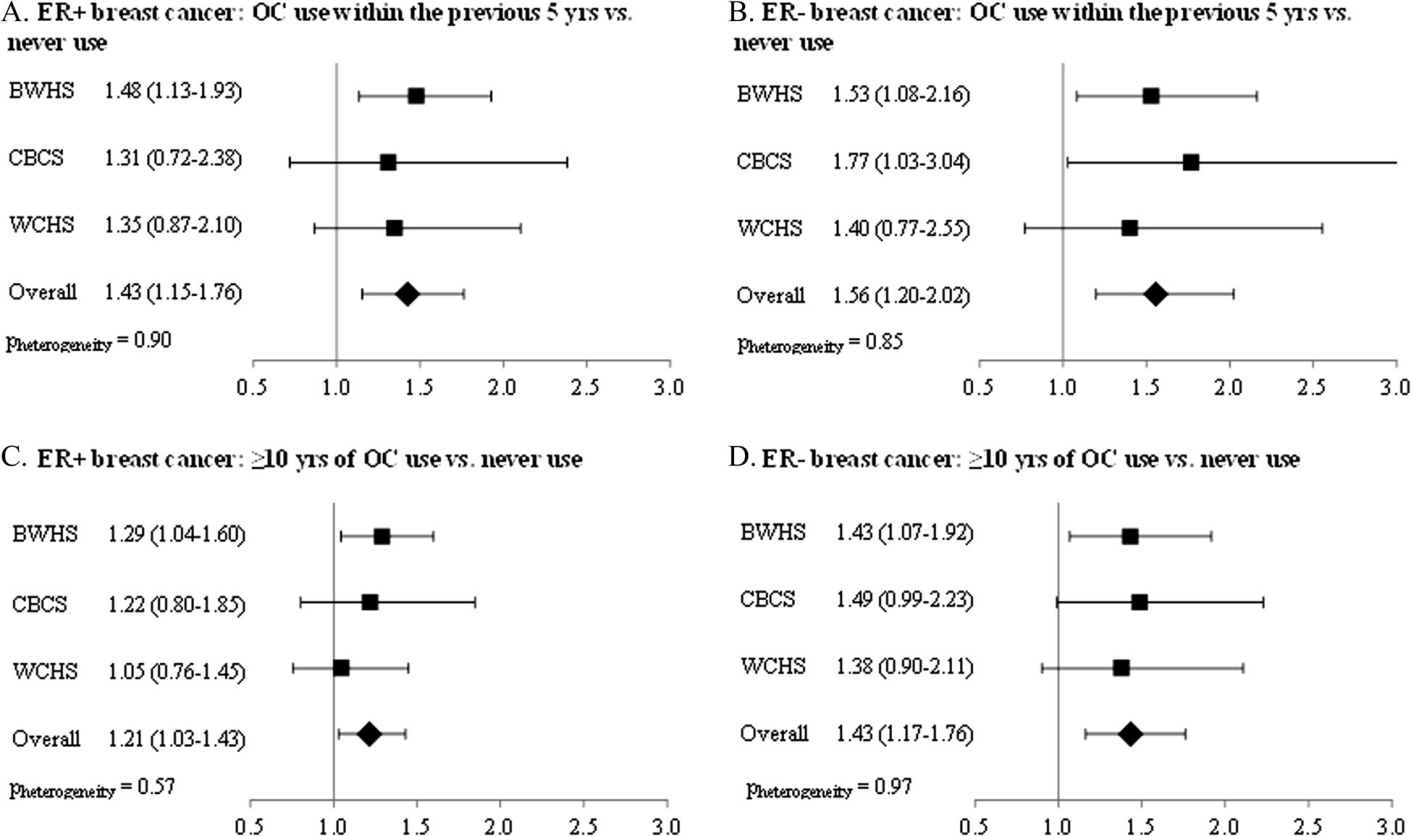
**Results from meta-analysis of oral contraceptive recency and duration in relation to breast cancer subtype.** Odds ratios and 95% confidence intervals from the full model by study and from a random-effects meta-analysis for oral contraceptive (OC) recency in relation to ER+ **(A)** and ER– **(B)** breast cancer and OC duration in relation to ER+ **(C)** and ER– **(D)** breast cancer. BWHS, Black Women’s Health Study; CBCS, Carolina Breast Cancer Study; ER, estrogen receptor; WCHS, Women’s Circle of Health Study.

Figure 1 presents study-specific estimates and ORs for recent (within previous 5 years) and long-term (≥10 years) OC use in relation to ER+ and ER– breast cancer computed from a meta-analysis with a random-effects model. The *P* value for heterogeneity was 0.57 or greater for all comparisons, indicating no statistically significant heterogeneity.

## Discussion

Both recent OC use and long duration of OC use were associated with increased risk of breast cancer in this study of African American women. The positive associations were observed for ER+, ER–, and TN breast cancer. ORs were highest for TN breast cancer: women who had used OCs in the past 5 years were estimated to have a 78% increased risk of TN breast cancer and those who had used OCs for at least 15 years had a 62% increase.

While some studies have found no association of recent OC use with overall breast cancer risk [2,3,5,38,39], many others have reported that recent OC users experience an increase in risk, which has varied from 24% to 60% [1,6,9,11,12,40]. The present findings are similar in magnitude to the previous positive associations. A collaborative analysis of 54 studies concluded that the increased risk of breast cancer associated with OC use disappears by 10 years after last use [1]. In a large case–control study that included African American women, no association was observed for long intervals (≥20 years) since last OC use [2,3]. However, in the present study of African American women, an increased risk was present up to 15 to 19 years after last use for ER+ cancer and after an even longer interval for ER– cancer. Results for duration of use are less consistent in the literature, with some studies reporting no association with long duration [2,3,25,39,41-43] while others observed a positive association [4-13]. In the present study, long duration use appeared to be more strongly associated with ER– cancer than with ER+ cancer.

There are few previous studies of OC use with molecular subtype of breast cancer. Five studies found associations of OC use with both ER+ and ER– breast cancer [4,8-10,12]. One study found an association for only ER+ breast cancer [24] and two found an association for only ER– breast cancer [23,44], while two other studies found no association for either subtype [45,46]. Of the studies that could evaluate TN or basal-like breast cancer, three found a positive association [10,23,26,47] while two others observed no association [2,25].

Combination OCs contain estrogens and progestins and have been found to increase circulating levels of estradiol [48,49]. A potential mechanism for increased risk of breast cancer with recent OC use is through estrogen-induced or progesterone-induced proliferation of breast cancer cells [50,51], which could cause progression of breast cancer. This mechanism may not be applicable for ER– breast cancer. Estrogens may also promote angiogenesis and stromal cell recruitment [52], which would be relevant for both ER+ and ER– breast cancer. OCs may also cause epigenetic changes, such as a decrease in DNA methylation [53]. DNA hypomethylation may be associated with tumor progression and metastasis [54] and represents another potential mechanism for the relation of OC use to breast cancer risk. It has been suggested that tumors regress after cessation of use of menopausal hormone supplements [55,56]. The tumors associated with menopausal hormone use are ER+. Whether tumor regression occurs after cessation of OC use is unknown, but this mechanism might not explain results for ER– breast cancer.

Few studies have examined potential effect modification of the relation of OC use to breast cancer risk by age [9,23,57]. Of these, some studies have observed a stronger association with risk among younger women than among older women, but this difference has generally not been statistically significant [4,26,58,59]. In a report from the BWHS, age did not significantly modify the association of OC use with breast cancer subtype [9]. In a Washington state case–control study, ORs for OC use were higher among women under age 40, but there was not a significant interaction by age [23]. In a multisite case–control study, ORs for OC use were significantly higher among women under age 35 and age 35 to 44 years, compared with women age 45 years and older [57]. In the present study, ORs were somewhat higher in the youngest age group, but there was not a significant interaction. ORs were also higher among premenopausal women than among postmenopausal women, but, again, the interaction was not statistically significant and there were few recent users among the postmenopausal women. Previous studies have observed associations of OC use with breast cancer risk in both premenopausal and postmenopausal women [8,45].

Differences in risk of breast cancer by age or menopausal status could reflect changes in OC formulations. The estrogen and progestin composition of OCs has varied, with declining doses of hormones and new types of progestin introduced over time [40,60,61]. There is no clear evidence that some formulations carry higher risk than others [5,62-64]. We were not able to evaluate formulation, as we did not have detailed information on OC type used. Thus, we cannot rule out the possibility that associations vary according to OC formulation. However, while younger women are more likely to use newer OC formulations, our findings did not vary significantly by strata of age or birth cohort in the present study. Moreover, estimates were similar across the studies, which were conducted in different time frames ranging from 1993 to present.

The associations of OC use with breast cancer risk in the present study were stronger among overweight and obese women. No previous studies have assessed whether an association of breast cancer risk with recent or long duration of OC use is modified by body mass index. This finding is particularly important given the high prevalence of obesity in African American women [65]. Obesity may alter the pharmacokinetic parameters of OCs, which could prolong exposure to estrogen in obese women due to an increase in the half-life of circulating estradiol, an increase in the rate of OC absorption, or an increase in bioavailability of the chemical compounds in the OCs [66-68].

The present analysis is the largest yet conducted of OC use in relation to specific subtypes of breast cancer among African American women. It allowed for informative assessment of associations within subtype overall and within some strata of interest. However, statistical power in some subanalyses was limited. Numerous important risk factors for breast cancer were controlled in the analyses. While the studies that contributed to the present analyses differed in study design and in population characteristics, a meta-analysis found no significant heterogeneity in the results. In addition, results on other risk factors, including parity and body size, have been similar across the individual studies [8,21,69-72]. Thus, the results are likely to be generalizable to other African American women. A limitation was that the classification by molecular subtype was based on the particular methods used in each pathology laboratory in the large number of hospitals from which the cases were derived. However, it is unlikely that misclassification will have been related to the OC use of cases. We did not have data to validate self-reported OC use, but validation studies have shown that OC use is generally well reported [5,73,74]. We did not have detailed information on OC type used, and therefore cannot rule out variations in associations by OC formulation.

## Conclusions

In sum, our findings suggest that recent OC use, particularly of long duration, is associated with increased risk of ER+, ER–, and TN breast cancer in African American women, with possibly a stronger relation with TN breast cancer. Increases in risk associated with OC use were apparent for up to 15 years or more after cessation of use. The association with OC use was most pronounced among overweight and obese women. Research is needed to investigate the mechanisms by which obesity may further increase the breast cancer risk associated with OC use.

## Abbreviations

AMBER: African American Breast Cancer Epidemiology and Risk
BWHS: Black Women’s Health Study
CBCS: Carolina Breast Cancer Study
CI: confidence interval
ER: estrogen receptor
HER2: human epidermal growth factor receptor 2
OC: oral contraceptive
OR: odds ratio
PR: progesterone receptor
TN: triple negative
WCHS: Women’s Circle of Health Study
